# A new way towards high-efficiency thermally activated delayed fluorescence devices via external heavy-atom effect

**DOI:** 10.1038/srep30178

**Published:** 2016-07-21

**Authors:** Wenzhi Zhang, Jiangjiang Jin, Zhi Huang, Shaoqing Zhuang, Lei Wang

**Affiliations:** 1 Wuhan National Laboratory for Optoelectronics, Huazhong University of Science and Technology, Wuhan 430074, China

## Abstract

Thermally activated delayed fluorescence (TADF) mechanism is a significant method that enables the harvesting of both triplet and singlet excitons for emission. However, up to now most efforts have been devoted to dealing with the relation between singlet-triplet splitting (ΔE_ST_) and fluorescence efficiency, while the significance of spin-orbit coupling (SOC) is usually ignored. In this contribution, a new method is developed to realize high-efficiency TADF-based devices through simple device-structure optimizations. By inserting an ultrathin external heavy-atom (EHA) perturber layer in a desired manner, it provides useful means of accelerating the T_1_ → S_1_ reverse intersystem crossing (RISC) in TADF molecules without affecting the corresponding S_1_ → T_1_ process heavily. Furthermore, this strategy also promotes the utilization of host triplets through Förster mechanism during host → guest energy transfer (ET) processes, which helps to get rid of the solely dependence upon Dexter mechanism. Based on this strategy, we have successfully raised the external quantum efficiency (EQE) in 4CzPN-based devices by nearly 38% in comparison to control devices. These findings provide keen insights into the role of EHA played in TADF-based devices, offering valuable guidelines for utilizing certain TADF dyes which possess high radiative transition rate but relatively inefficient RISC.

In recent years, the interest in synthesizing thermally activated delayed fluorescence (TADF) molecules has grown tremendously due to their potentials for obtaining nearly 100% internal quantum efficiency (IQE)^1^,^2^,^3^,^4^,^5^,^6^,^7^. In TADF process, it is reported that light emission is enhanced through efficient up-conversion from the lowest triplet excited states (T_1_) to the lowest singlet excited states (S_1_), if the singlet-triplet energy splitting (ΔE_ST_) of the molecule is sufficient small (≤100 meV) to increase their first-order mixing coefficient^8^,^9^. However, specific details of the emission mechanism remain to be further investigated.

For the purpose of a high rate constant of reverse intersystem crossing (k_RISC_), TADF molecules usually possess a large spatial separation of the frontier orbitals to achieve a remarkably small ΔE_ST_. Unfortunately, such a molecule design strategy usually results in a low radiative transition rate constant of singlet states (k_r_) according to Fermi’s golden rule^8^,^9^. Up to now, extensive efforts have been devoted to dealing with this inherent contradiction, including fine-tuning the delocalization of the frontier orbitals in a TADF compound to enhance k_r_ without increasing ΔE_ST_^10^, cutting off the relevance of k_RISC_ and k_r_ by innovatively utilizing TADF materials as sensitizing hosts^11^,^12^,^13^, and putting forward a new hybridized local and charge-transfer (HLCT) theory^14^,^15^. Noteworthy, these strategies have only paid a little attention to the influence of spin-orbit coupling (SOC) on TADF even though efficient RISC is also reported to be possible in particular Sn^4+^−porphyrin and Cu(I) complexes. Actually, the major difference of these heavy-metal complexes compared to all-organic molecules is the stronger spin-orbit coupling (SOC) introduced by their internal heavy-atom (IHA)^16^,^17^, which, on the basis of Fermi’s golden rule and Frank-Condon principle, has an appreciable effect on the rate constants of both intersystem (k_ISC_) and reverse intersystem (k_RISC_) crossing^18^. Considering the similarity between IHA effect and its analogue external heavy-atom (EHA) effect, we assume that the TADF process can also be fine-tuned by utilizing a proper EHA. That is, by manipulating the degree of EHA effect through a device-structure optimization, we suppose that the enhancement of k_RISC_ can be comparable to or even larger than that of k_ISC_ in the presence of stronger SOC, and then higher luminescence efficiency is worth looking forward to.

Besides, a further improvement of host → guest energy transfer (ET) is another formidable challenge. Due to the high polarity of most TADF molecules, an ordinary host-guest configuration, i.e., dispersing TADF dyes into conventional fluorescent host materials at a low doping concentration, is employed to alleviate some detrimental exciton-exciton interactions^8^,^9^. It is generally accepted that singlet excited states of host materials (S_1_^H^) can transfer to singlet excited states of TADF guests (S_1_^G^) following the resonant Förster process, while the detailed ET mechanism between host triplet excitons (T_1_^H^) and guest molecules is not clarified. Considering the limitation of spin conservation, a T_1_^H^ → T_1_^G^ Dexter ET between hosts and guests is believed to be a dominant approach to utilize host triplet excitons in metal-free OLEDs. However, the aforementioned low doping concentration of TADF molecules can be a main obstacle to the short-range Dexter ET. A lot of methods have been gradually proposed to reduce the energy loss resulted from dark host triplet excitons. The first one is facilitating a direct charge recombination on TADF dyes^19^,^20^. Unfortunately, a relatively high doping concentration is usually employed to ensure an efficient direct charge-trapping, which usually yields intrinsic triplet-charge annihilation (TCA) or aggregate-induced quenching in the emissive layer (EML)^21^,^22^,^23^. Another method is utilizing TADF dyes as assistant dopants along with conventional fluorescence emitters^24^, in which an ideal result is obtained on the basis of following premises, i.e., 1) the formation of fluorescence emitter triplet excitons should be avoided, no matter they are resulted from direct charge-trapping on conventional fluorescence emitters or from triplet-triplet Dexter ET; 2) the potential energy loss caused by triplet excitons quenching of TADF molecules should also be prevented. Despite these advances to date, a further investigation is all along necessary. Enlightened by a common phosphor-sensitization technology which is popular in hybrid white OLEDs^4^,^25^,^26^,^27^,^28^,^29^, in this contribution we suppose that a simple but important consideration for promoting host → guest ET is taking advantage of EHA effect. With the aid of EHA perturber, the host triplet excitons can efficiently transfer to guest molecules via an unusual T_1_^H^ → S_1_^H^ Förster ET mechanism, which gives a better opportunity to simultaneously harvest both triplet and singlet excitons of host materials.

Based on the previous considerations, we put forward a new device-structure optimization strategy towards high efficiency by introducing EHA into TADF-based devices. A blue phosphorescent Bis(3,5-difluoro-2-(2-pyridyl)phenyl-(2-carboxypyridyl) iridium (FIrpic) was chosen as a perturber in our experiment since its EHA effect had been detailed studied in a lot of white OLEDs incorporating phosphor-sensitization mechanism^30^,^31^. Meanwhile, 1,2,3,4-tetrakis(carbazol-9-yl)-5,6-dicyanobenzene (4CzPN) was chosen as a TADF guest material in this case. By means of detailed photoluminescence (PL) decay and photoluminescence quantum yield (PLQY) measurements, it was demonstrated that the introduction of iridium atom accelerated the T_1_ → S_1_ RISC process of 4CzPN while its S_1_ → T_1_ ISC process was scarcely changed in the presence of EHA. Meanwhile, the ET between T_1_^H^ states of hosts and S_1_^G^ states of 4CzPN was also promoted through an unusual Förster mechanism with the aid of FIrpic^25^,^32^. That is, by adding a proper perturbation in symmetry via an EHA perturber, the non-radiative mode of host triplet excitons can be counteracted and therefore yields efficient T_1_^H^ → S_1_^G^ ET from hosts to emitters^25^. Since the Förster ET is a long-range process, the ET efficiency will no more subject to the relative low doping concentration of TADF dyes. Concretely speaking, in optimized 2,5-Bis(2-(9H-carbazol-9-yl)phenyl)-1,2,4-thiadiazole-based (*o*-CzTHZ-based) OLEDs with an ultrathin FIrpic layer, the external quantum efficiency (EQE) is enhanced by a factor of about 1.3~1.4 in comparison with those of control ones, and a maximum forward-viewing EQE of 17.9% is achieved. Such a device-structure optimization is attractive not only for providing keen insights into the role of EHA played in TADF-based devices, but also for offering valuable guidelines for proper and full utilization of certain TADF dyes which possess relatively inefficient RISC but high k_r_.

## Results

As is well understood, the quantum efficiency of an OLED is mainly managed by two factors: the intrinsic luminescence efficiency of guest molecules and the host → guest ET efficiency in the EML. In general, most TADF dyes suffer an inherent contradiction between k_r_ and k_RISC_ due to a large spatial separation of their frontier orbitals. This contradiction is detrimental to the luminescence efficiency of TADF dyes even though a compromise have been made between k_r_ and k_RISC_. Moreover, the triplet excitons of fluorescent host materials cannot be efficiently utilized through T_1_^H^ → T_1_^G^ Dexter ET process since a relative low doping concentration of TADF guests is usually presented. Therefore, these factors may be obstacles for higher devices performances.

**Figure 1:**
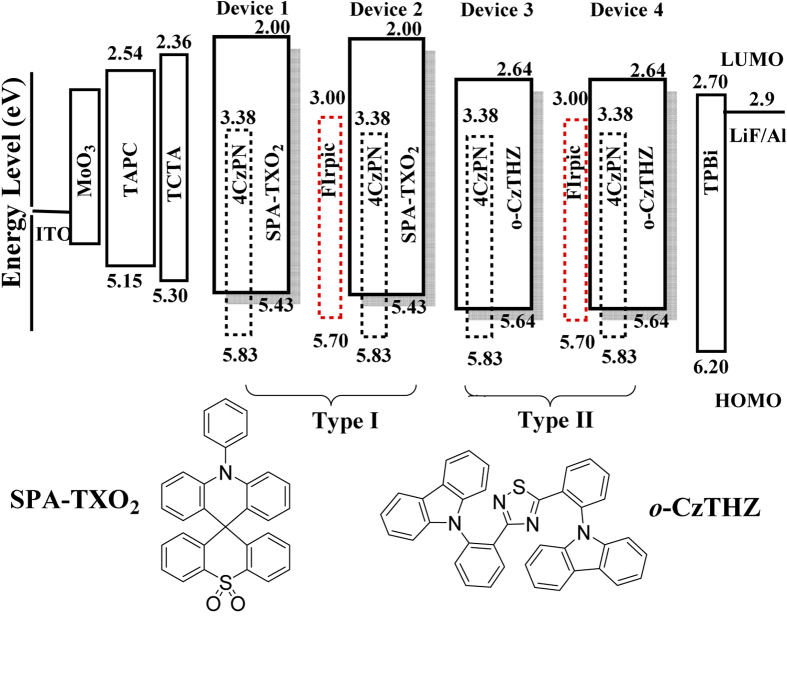
The configurations of all 4CzPN-based devices and energy level diagrams of all the materials used, including the molecule structures of SPA-TXO_2_ and *o*-CzTHZ.

In the following investigations, four optimized OLEDs with green TADF material 4CzPN and deep blue FIrpic were fabricated to explore the feasibility of using an EHA perturber to improve device performances, with the simple configuration of ITO/MoO_3_ (8 nm)/TAPC (60 nm)/TCTA (5 nm)/FIrpic (0 or 0.5 nm)/EML (25 nm)/TPBi (60 nm)/LiF (0.5 nm)/Al (100 nm) (As described in Fig. 1). According to the different triplet energy levels (E_T_) of the host materials, these devices could be broadly divided into two types. Specifically, device **1** in type **I** was hosted by 10-phenyl-10*H*-spiro(acridine-9,9′-thioxanthene) 10′,10′-dioxide (SPA-TXO_2_) with a higher E_T_ (3.08 eV) and the concentration of 4CzPN dyes was optimized to be 3 wt%^33^, whereas in device **2** an additional FIrpic was inserted as an isolated ultrathin-layer between EML and hole transport layer (HTL) instead of common co-doped configuration^25^,^26^, to cut the potential risk of forming additional recombination centers. As a comparison, device **3** and **4** in type **II** were hosted by *o*-CzTHZ with a lower E_T_ (2.62 eV).

**Figure 2:**
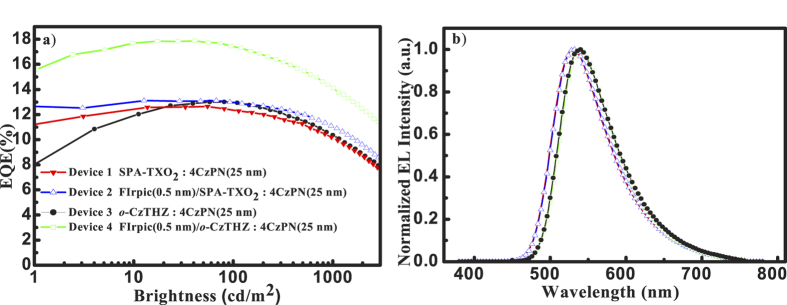
Electroluminescence properties of all devices. (**a**) EQE-Brightness characteristics; (**b**) The EL spectra measured at about 100 cd/m^2^.

The EQE-brightness curves of all devices are presented in Fig. 2(a) and detailed data are summarized Table 1. In concrete terms, maximum EQE up to 17.9% is achieved in device **4** and the relative EQE enhancement between in device **3** and **4** is up to 38%. On the contrary, there is no obvious change between device **1** and **2** and the EQEs in devices in type **I** are always lower than the related ones in type **II**. Meanwhile, all the devices exhibit similar EL emission arising from 4CzPN, which excludes other emission sources. Noteworthy, the isolated FIrpic layer was also inserted between EML and electron transport layer (ETL) in *o*-CzTHZ-based devices, as well as in the middle of two separated thin EML. The efficiency enhancement shown in the latter case is still obvious, while the result in the former case is not satisfactory especially among the high current range (see Supplementary Figure S1). We attribute that increased efficiency roll-off to the potential formation of additional recombination centers on FIrpic. As a consequence, detailed investigation should be conducted to comprehend the different roles of EHA perturber in different cases, especially in terms of the two factors abovementioned.

## Discussion

Considering the fact that a change of the hole transport will lead to a change of the charge recombination in the EML, we initially fabricated four hole-only devices (HODs) to give a quantitative investigation of the potential variation in hole transport ability^34^. Noteworthy, the current density-voltage (J-V) characteristics change little in both SPA-TXO_2_-based and *o*-CzTHZ-based HODs after inserting an isolate FIrpic layer (see Supplementary Figure S2), which excludes the influence of FIrpic on charge recombination under electrical excitation. Besides, it also excludes the formation of interfacial charge transfer (CT) state between FIrpic and 4CzPN since the hole cannot be trapped on FIrpic molecules in above four cases. That is to say, an extrafluorescent electroluminescence^35^ should be extremely weak in this contribution.

To further understand the origin of the EQE enhancement, initially it is crucial to examine the influences of EHA on the intrinsic luminescence efficiency of TADF molecules, especially on both S_1_ → T_1_ ISC and T_1_ → S_1_ RISC cycles^36^. Berberan-Santos *et al*. has preliminary reported that the introduction of EHA perturber significantly improved the ISC and RISC process of C70, and he attributed the special phenomenon to the small ΔE_ST_ of C70^37^. Considering the smaller ΔE_ST_ of ordinary donor-acceptor molecules, we assume that a similar phenomenon can be also observed in TADF materials. Moreover, since there is a competition between the ISC and radiative transition process of singlet states, we speculate that the ISC process may be slightly affected by EHA if the corresponding k_r_ is much higher than k_ISC_.

**Figure 3:**
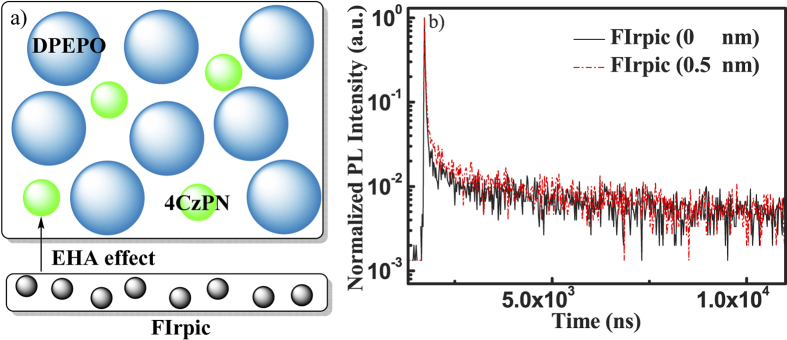
(**a**) Configurations of thin films consisting of DPEPO: 3 wt% 4CzPN (film 1, 40 nm) and FIrpic (0.5 nm)/DPEPO: 3 wt% 4CzPN (film 2, 40 nm); (**b**) Transient behaviors of 4CzPN in film 1 and 2, measured under ambient conditions and observed at 530 nm.

Two films containing bis[2-(diphenylphosphino)phenyl]ether oxide (DPEPO): 3 wt% 4CzPN with (film **1**, see Fig. 3, 40 nm) and without (film **2**, 40 nm) an ultrathin FIrpic layer (0.5 nm) were fabricated, and their solid-state PLQYs were measured, respectively. On one hand, the photo-excitation excluded the interferences of charge-trapping and charge balance in electroluminescence (EL) devices. On the other hand, the excitation wavelength could be fixed at 460 nm to exclude the influences of ET from hosts to guests, by alleviating the possibility of exciting host matrix (DPEPO) and EHA perturber (FIrpic). As we expected, the solid-state PLQY is increased from 45% to 55%, by adding 0.5 nm FIrpic layer.

Furthermore, the micro process of ISC and RISC could be directly examined through detailed PL decay measurement and analyses. The transient PL decay characteristics of 4CzPN were further observed at 530 nm in both samples, and the decay curves were well fitted by three-exponentials. According to the definition 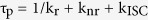 (1), the prompt fluorescence lifetime (τ_p_) is determined by the radiative (k_r_) and non-radiative (k_nr_) rate constant of S_1_ states, as well as k_ISC_. As is well understood in phosphorescent materials, the EHA can increase k_ISC_ by enhancing the SOC between S_1_ and T_1_ states, while has little effect on both k_r_ and k_nr_ since the spin is not changed during the S_1_ → S_0_ decay process. Therefore, we assume that the change of τ_p_ calculated from prompt decay component of the decay curves reflects the change of k_ISC_.

As shown in Table 2, the τ_p_ of 4CzPN are almost the same in both two films, indicating that k_ISC_ of 4CzPN is slightly affected in this case. On the contrary, a moderate decrease of the delayed fluorescence lifetime (τ_d_) is observed from film **1** to film **2**, mainly owing to the enhanced S_1_^G^ → T_1_^G^ RISC of 4CzPN guests in the presence of higher SOC in film **2**. Detailed information about k_RISC_ and k_ISC_ is shown in Supplementary Table S1. What’s more, a further increase of k_RISC_ may be still possible if we fine-tune the degree of EHA effect by using other perturbers or different device structures. Further investigations are still necessary.

In addition, the influence of EHA on host → guest ET processes can be another key in determining EQE enhancement. Direct evidences are provided by comparing the EQE and EL spectra of devices with and without using FIrpic. As aforementioned, the relative EQE enhancement between in device **3** and **4** is up to 38%, while there is no obvious change between in device **1** and **2**. Meanwhile, all the devices exhibit similar EL emission arising from the 4CzPN, which excludes other emission sources. Since the host matrix is the only difference between type **I** and **II**, we ascribed the discrepancy in EQE enhancement to a possible difference in their host → guest ET routes.

Herein, absolute PLQYs in all different films (from film **3** to film **8**) were also measured and the excitation wavelength was fixed at 280 nm to excited both SPA-TXO_2_ and *o*-CzTHZ. Since the doping concentration of 4CzPN is identical in all films, it is believed that the variation trends of PLQYs shown in Table 3 can directly reflect the variation trends of ET efficiencies. Initially, we believe that S_1_^H^ → S_1_^G^ ET via Förster mechanism cannot be the sole route for host → guest ET in film **3** and **6** in absence of EHA. Actually, the spectral overlap between the emission profile of SPA-TXO_2_ and absorption profile of 4CzPN at room-temperature (RT) is larger than that between *o*-CzTHZ and 4CzPN (see Supplementary Figure S3). Meanwhile, a better radiative decay of S_1_^H^ states is also observed in pure SPA-TXO_2_ film by measuring the solid-state PLQYs (8.4% for SPA-TXO_2_ while 2.3% for *o*-CzTHZ, excited at 280 nm). According to Förster theory, it is easy to predict a more efficient ET between SPA-TXO_2_ and 4CzPN without the help of EHA. However, the actual result is contrary to our expectations no matter in EL devices (device **1** and **3**) or PL films (film **3** and **6**). Thus, we ascribe the contradiction to the potential different utilization rate of the T_1_^H^ excitons. In addition, with the growth of the thickness of the FIrpic layer, a stable enhancement in PLQYs is achieved in 4CzPN-based films hosted by *o*-CzTHZ, while PLQYs in films hosted by SPA-TXO_2_ show less sensibility to EHA. Considering the different E_T_ levels of SPA-TXO_2_ and *o*-CzTHZ, the discrepancy implies that the utilization route of T_1_^H^ excitons during host → guest ET processes can be influenced by additional SOC effect, and the influence is related to the E_T_ levels of host materials.

**Figure 4:**
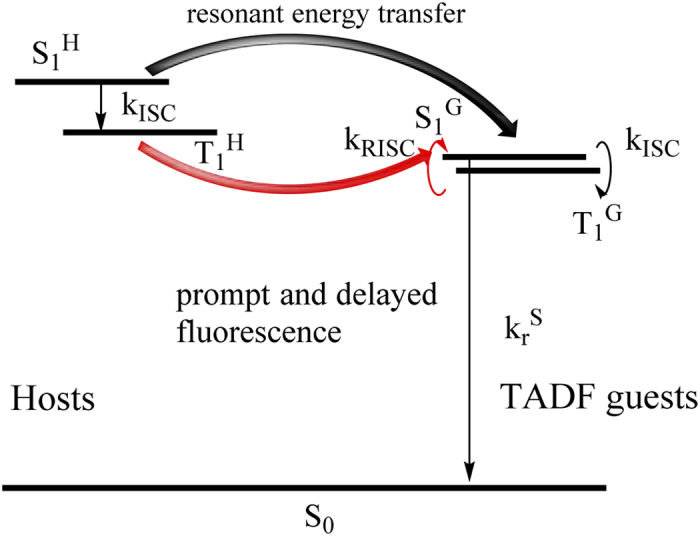
A proposed T_1_ → S_1_ energy transfer route from hosts to TADF guests and enhanced RISC process of TADF guests, with the aid of EHA.

According to aforementioned analyses, we newly propose a potential T_1_^H^ → S_1_^G^ ET from host to guest through resonant Förster process^38^,^39^, i.e., ^3^D^*^ + ^1^A → ^1^D + ^1^A^*^, as shown in Fig. 4. Here, D represents donor and A represents acceptor. Triplet and singlet states are signified by a superscript 3 or 1, respectively, and excited states are marked by asterisks. Initially, the large spectral overlap between the low-temperature (LT) emission profile of *o*-CzTHZ and the absorption profile of 4CzPN (see Supplementary Figure S3) ensures the feasibility of this transfer according to Förster theory. In addition, the spin is changed during this ET process, implying that such a process can be fine-tuned by additional SOC effect. In this case, by adding FIrpic as a proper EHA perturber, the non-radiative mode of T_1_^H^ state of *o*-CzTHZ is changed and the oscillator strength of its radiative decay is increased^40^. Thus, both two prerequisites of the T_1_^H^ → S_1_^G^ ET through Förster process are satisfied in our experiments, and the experimental phenomena are in well accordance with the theoretical analyses. Noteworthy, such facilitated ET in type **II** also cannot be ascribed to a conventional phosphor-sensitization technology^25^. Firstly, according to the higher E_T_ of FIrpic than that of *o*-CzTHZ, FIrpic cannot act as a bridge in the ET processes between *o*-CzTHZ and 4CzPN molecules. Secondly, both LUMO and HOMO level of FIrpic are shallower than those of 4CzPN, respectively, which excludes the formation of FIrpic excitons. In short, the FIrpic is purely used as an EHA perturber layer here and the device efficiency is partly enhanced through a direct T_1_^H^ → S_1_^G^ ET rather than an cascade one which is popular in phosphor-sensitization technology. What’s more, considering to the larger difference between T_1_^H^ level of SPA-TXO_2_ and S_1_^G^ level of 4CzPN, we assume that this ET process is also related to the energy levels. Specifically, a huge energy difference between T_1_^H^ and S_1_^G^ can be an obstacle to effective T_1_^H^ → S_1_^G^ ET, even though the EHA perturber is present.

In summary, by introducing heavy metal through manipulating device architectures, a simple but robust method towards high-efficiency TADF devices has been exploited. On one hand, by enhancing SOC through EHA effect, the T_1_ → S_1_ RISC process of TADF dyes was demonstrated to be accelerated without affecting its S_1_ → T_1_ process heavily, especially for several TADF molecules possessing high PLQY. On the other hand, a T_1_^H^ → S_1_^G^ ET process through Förster mechanism was newly found in conventional TADF-based host-guest systems, which could be also facilitated with the aid of EHA. Therefore, a maximum EQE of 17.9% was achieved in a 4CzPN-based device inserted by ultrathin EHA layer, which is increased by up to 38% in comparison to that in the control device. Generally, our research provides keen insights into the role of EHA played in TADF-based devices, opening a new avenue for efficiently utilizing TADF dyes which possess high k_r_ but relatively inefficient RISC.

## Methods

### OLEDs Fabrication and Measurement

Like our other researches^41^,^42^, all the devices were fabricated upon the surface of indium-tin-oxide (ITO)-coated glass substrates. The sheet resistance of these substrates was approximately 25 Ωsquare^−1^. In fabrication procedure, the glass substrates were exposed to oxygen plasma for 5 min after sequential chemical cleaning and drying. Then they were loaded into a high vacuum (under a base pressure ca. 5 × 10^−6^ Torr) thermal evaporation chamber, followed by successive deposition at a rate of 0.9–1.1 Å/s. In addition, the EML was co-evaporated and the deposition rate of the dopants were precisely controlled by individual quartz-crystal thickness monitors according to the doping fraction. The devices structure was optimized to ITO/molybdenum trioxide (MoO_3_, 8 nm)/1,1′-bis (di-4-tolylaminophenyl) cyclohexane (TAPC, 60 nm)/1,4,7-tris(acetato)-1,4,7-triazacyclononane (TCTA, 5 nm)/Bis(3,5-difluoro-2-(2-pyridyl)phenyl-(2-carboxypyridyl) iridium (Firpic, 0 or 0.5 nm)/EML (25 nm)/1,3,5-tris(2-N-phenylbenzimidazolyl) benzene (TPBi, 60 nm)/lithium fluoride (LiF, 0.5 nm)/Aluminium (Al, 100 nm). The performances, including Current density (*J*)–voltage (*V*)–luminance (*L*) characteristics, electroluminescent (EL) spectra, luminance, Commission Internationale de l’Eclairage (C.I.E.) coordinates as well as various efficiencies of the devices were measured by combining the Keithley 2400 Digital Source meter with the PR655 at room temperature. UV-Vis absorption spectra were recorded on a Shimadzu UV-VIS-NIR Spectrophotometer (UV-3600). The steady-state PL characteristics were measured using Edinburgh instruments (FLS920 spectrometers) and the transient PL decay behaviors were recorded using a Quantaurus-Tau fluorescence lifetime measurement system (C11367-01, Hamamatsu Photonics). Absolute PLQYs of were obtained using Quantaurus-QY measurement system (C11347-11, Hamamatsu Photonics).

## Additional Information

**How to cite this article**: Zhang, W. *et al*. A new way towards high-efficiency thermally activated delayed fluorescence devices via external heavy-atom effect. *Sci. Rep.* **6**, 30178; doi: 10.1038/srep30178 (2016).

## Supplementary Material

Supplementary Information

## Figures and Tables

**Table 1:** The EL properties of all devices.

Device	Host	Thickness of FIrpic [nm]	V_on_ [V]	[η_c_]^a^[cd/A]	[η_p_]^a^[lm/W]	[η_EQE_]^a^[%]	CIE [x, y]^b^
**1**	SPA-TXO_2_	0	2.95	41.8	40.7	12.6	(0.35, 0.59)
**2**	SPA-TXO_2_	0.5	2.95	43.5	42.6	13.1	(0.35, 0.59)
**3**	*o*-CzTHZ	0	3.20	44.1	38.1	13.0	(0.38, 0.58)
**4**	*o*-CzTHZ	0.5	3.30	61.1	50.5	17.9	(0.38, 0.58)

Von: Turn-on voltage at 1 cd/m^2^. η_c_: Current efficiency. η_p_: Power efficiency. η_EQE_: External quantum efficiency. ^a^Maximum. ^b^Measured at 100 cd/m^2^.

**Table 2:** The absolute solid-state PLQYs and transient PL decay characteristics of 4CzPN in film 1 and 2.

Film	Thickness of FIrpic [nm]	τ_p_ [ns]	τ_d_ [μs]	PLQY [%]
**1**	0	16.02	6.86	45
**2**	0.5	16.83	5.10	55

τ_p_: prompt fluorescence lifetime; τ_d_: delayed fluorescence lifetime calculated using 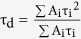, where A_i_ is the pre-exponential for lifetime τ_i_.

**Table 3:** The absolute PLQYs in films from 3 to 8.

Film	Host	Thickness of FIrpic [nm]	PLQY [%]
**3**	SPA-TXO_2_	0	58.3
**4**	SPA-TXO_2_	0.5	60.1
**5**	SPA-TXO_2_	1	60.5
**6**	*o*-CzTHZ	0	82.5
**7**	*o*-CzTHZ	0.5	85.2
**8**	*o*-CzTHZ	1	91.2
