# Supplementary material for: A new way towards high-efficiency thermally activated delayed fluorescence devices via external heavy-atom effect
Source: Sci Rep. 2016 Jul 21;6:30178. doi: 10.1038/srep30178 (PMC4954963; doi:10.1038/srep30178)
Supplement: Supplementary Information [file srep30178-s1.pdf]

A new way towards high-efficiency thermally activated delayed fluorescence devices via external heavy-atom effect

Wenzhi Zhang, Jiangjiang Jin, Zhi Huang, Shaoqing Zhuang, and Lei Wang*
Wuhan National Laboratory for Optoelectronics, Huazhong University of Science and Technology, Wuhan 430074, China
*wanglei@mail.hust.edu.cn

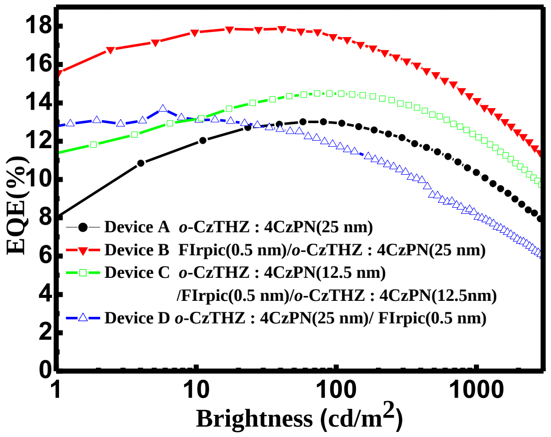

Figure S1. EQE-Brightness characteristics of other four devices, in which the FIrpic layers were inserted at different locations. The device structure is ITO/MoO_3 (8 nm)/TAPC (60 nm)/TCTA (5 nm)/ EML (x nm)/FIrpic (0 nm for device A or 0.5 nm for the others)/EML (25-x nm)/ TPBi (60 nm)/LIiF (0.5 nm)/Al (100 nm), x=0 nm, 12.5 nm, 25 nm for device B, C, and D, respectively.

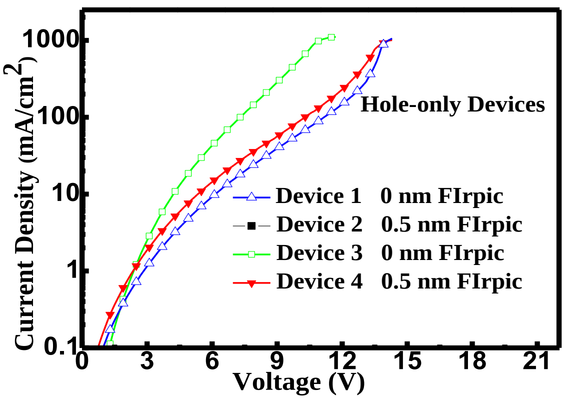

Figure S2. Current density-voltage (J-V) characteristics of four hole-only devices, with the structure of ITO/MoO_3 (8 nm)/TAPC (60 nm)/TCTA (5 nm)/FIrpic (0 or 0.5 nm)/EML (25 nm)/ MoO_3 (8 nm)/Al (100 nm). The structures of the FIrpic layer and EMLs are as the same as those in related EL devices from **1** to **4**.

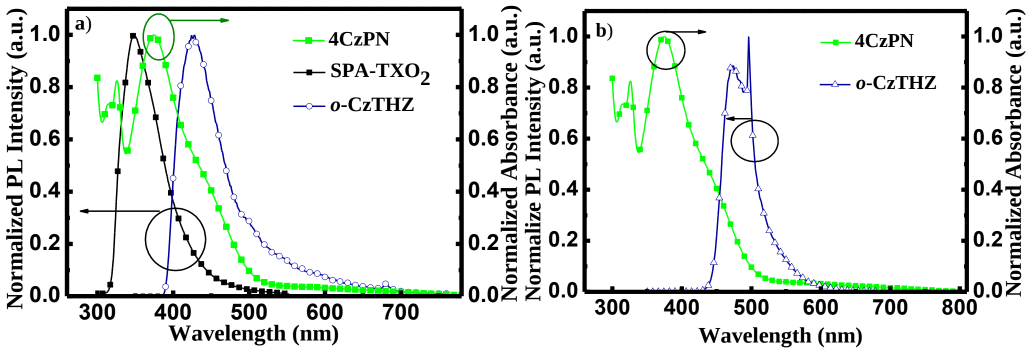

Figure S3. a) UV/Vis absorption spectra of 4CzPN in neat film, and PL spectra of SPA-TXO_2 and *o*-CzTHZ in neat films at RT; b) UV/Vis absorption spectra of 4CzPN in neat films and PL spectra of *o*-CzTHZ in 2-methyltetrahydrofuran at 77 K.

Table S1. The Rate constants of 4CzPN in film 1 and 2

Film	Thickness of FIrpic [nm]	k_p [s^{-1}]	k_d [s^{-1}]	k_{ISC} [s^{-1}]	k_{RISC} [s^{-1}]
**1**	0	$1.62*10^7$	$3.73*10^4$	$4.61*10^7$	$0.96*10^4$
**2**	0.5	$1.19*10^7$	$5.10*10^4$	$4.76*10^7$	$2.23*10^4$

k_p:rate constant of the prompt fluorescence component; k_d: rate constant of the delayed fluorescence component; k_{ISC}: rate constant of intersystem crossing; k_{RISC}: rate constant of reverse intersystem crossing. All rate constants are calculated by using 4 formulas: (1) $k_p = \frac{\Phi_p}{\tau_p}$; (2) $k_d = \frac{\Phi_d}{\tau_d}$; (3) $\Phi_p = \frac{k_p}{k_p + k_{ISC}}$; (4) $k_{RISC} = \frac{k_p \times k_d}{k_{ISC}} \times \frac{\Phi_d}{\Phi_p}$; Φ_p is the photoluminescence quantum efficiency of the prompt component, Φ_d is the photoluminescence quantum efficiency of the delayed component.
